# Bayesian multilevel modelling of the association between socio-economic status and stunting among under-five-year children in Tanzania

**DOI:** 10.1186/s41043-023-00474-3

**Published:** 2023-11-29

**Authors:** Edwin Musheiguza, Tukae Mbegalo, Justine N. Mbukwa

**Affiliations:** 1 Department of Mathematics and Information Communication Technology, College of Business Education, P.O. Box 1968, Dar es Salaam, Tanzania; 2 Department of Mathematics and Statistics Studies, Mzumbe University, P.O Box 87, Mzumbe, Tanzania

**Keywords:** Stunting, Bayesian multilevel generalized linear mixed models, Tanzania

## Abstract

**Background:**

Stunting is associated with socioeconomic status (SES) which is multidimensional. This study aimed to compare different SES indices in predicting stunting.

**Methods:**

This was the secondary data analysis using Tanzania Demographics and Health Surveys (TDHS). The study used 7492, 6668, and 8790 under-five-year children from TDHS 2004/5, 2010, and 2015/16, respectively. The Household Wealth Index (HWI); Water and Sanitation, Assets, Maternal education and Income (WAMI); Wealth Assets, Education, and Occupation (WEO); and the Multidimensional Poverty Index (MPI) indices were compared. The summated scores, principal component analysis (PCA), and random forest (RF) approaches were used to construct indices. The Bayesian and maximum likelihood multilevel generalized linear mixed models (MGLMM) were constructed to determine the association between each SES index and stunting.

**Results:**

The study revealed that 42.3%, 38.4%, and 32.4% of the studied under-five-year children were stunted in 2004/5, 2010, and 2015/16, respectively. Compared to other indicators of SES, the MPI had a better prediction of stunting for the TDHS 2004/5 and 2015/16, while the WAMI had a better prediction in 2010. For each score increase in WAMI, the odds of stunting were 64% [BPOR = 0.36; 95% CCI 0.3, 0.4] lower in 2010, while for each score increase in MPI there was 1 [BPOR = 1.1; 95% CCI 1.1, 1.2] times higher odds of stunting in 2015/16.

**Conclusion:**

The MPI and WAMI under PCA were the best measures of SES that predict stunting. Because MPI was the best predictor of stunting for two surveys (TDHS 2004/5 and 2015/16), studies dealing with stunting should use MPI as a proxy measure of SES. Use of BE-MGLMM in modelling stunting is encouraged. Strengthened availability of items forming MPI is inevitable for child growth potentials. Further studies should investigate the determinants of stunting using Bayesian spatial models to take into account spatial heterogeneity.

## Introduction

Over decades, stunting among under-five-year children has been a global health concern. Stunting is defined as poor linear growth among under-five-year children which hinders child growth potentials [1, 2]. Stunting leads to poor participation in production activities and academic performance due to reduced school attendance [3] and accelerates repeated infections [4]. Furthermore, stunting leads to deaths and injuries [5] which in turn increases health spending among the economically disadvantaged population. Compared to other forms of undernutrition, stunting is the general indicator of child’s overall levels of undernutrition and poor life quality [6]. Additionally, compared to other forms of undernutrition, the observed higher prevalence of stunting at both global and national levels over decades [7] requires further investigation.

Globally, it was estimated that 149.2 (22%) million under-five-year children were stunted in 2021 [7]. In 2017, approximately 2% of 155 million stunted children lived in high-income countries while about 50% lived in low-income countries [8]. Out of 155 million stunted children in 2017, about 59 million lived in Africa of which 24 million lived in Eastern Africa [8]. These figures imply that despite global efforts to reduce stunting, progress has been uneven and slow, especially in low-income countries and regions. Tanzania, being one such country, experiences a high burden of stunting that varies across regions and districts. In Tanzania, stunting declined too slowly from 34 to 31.8% in 2016 and 2018, respectively [9]. According to the WHO-UNICEF classification [7], the prevalence of stunting in Tanzania was very high (above 30%) in 26 regions and above 40% in 6 regions [9]. In these aspects, we should not be comfortable with the overall trend of stunting at a national level, as it conceals the larger disparity within and between regions. The observed uneven distribution of stunting is underlined by the fact that diseases occur at random and have different causal pathways, triggered by interaction between socioeconomic factors [10–12].

Different SES indices for predicting health outcomes have been developed including single indices like maternal education [10, 13–15], income [10, 15, 16] and multivariate indices like wealth assets [13, 17, 18], WEO [12, 15], WAMI and MPI [13, 15]. Health outcomes including stunting do not occur at random, as socioeconomic determinants are unevenly distributed. The fact that SES variables interact differently when causing diseases, necessitates the establishment of the best indicator of SES associated with stunting rather than using the known measure of SES (HWI). Knowing the SES index associated with stunting could be useful for developing targeted and focused interventions in developing countries including Tanzania.

The mostly used proxy of SES at the household level is the Household Wealth Index (HWI) [10, 13, 19–22]; however, this index does not include mother’s education [12, 13, 21] and occupation [12] which are important indicators of SES. Other indices include the availability of safe Water and sanitation, Assets, Maternal education and Income (WAMI) and the Multidimensional Poverty Index (MPI) [13]. The multicountry study conducted in Africa assessing the measurement of socioeconomic status in relation to stunting revealed that the WAMI index had a statistically significant negative relationship with stunting [13]. The HWI and maternal education had a negative association with stunting, as children from poor-wealth households and born from uneducated mothers had higher odds of being stunted as compared to children from highest-wealth quintile households [19, 21, 23–31]. In Tanzania, most studies use HWI as an indicator of SES [27, 32–36], ignoring the possible correlation between HWI and other indicators of SES including occupation and maternal education. However, the findings that WAMI predicted stunting better than maternal education, HWI and MPI [13] were limited to children living in Haydom district, Manyara region, hence lacking generalizability to the whole country. Furthermore, the study [13] did not compare the WEO with other SES indices.

Furthermore, despite the available advanced statistical modelling techniques of child stunting like the multilevel generalized linear mixed models (MGLMM) still are not sufficiently utilized in Tanzania. Most studies use classical regression models (logistic and linear) [28, 36–43] and very little on the MGLMM [32] which use the maximum likelihood estimation (MLE) techniques. The multilevel models take into account unmeasurable factors [44, 45] and control the correlation between subjects and geographical areas using random effects [32]. However, compared to MLE multilevel generalized linear mixed models (MLE-MGLMM), the Bayesian Estimation -MGLMM (BE-MGLMM) produces unbiased estimates even in areas with smaller sample sizes [46, 47]. Additionally, the BE-MGLMM produces unbiased estimates even at level two where the MLE- MGLMM may lead to biased estimates [48]. The fact that mother–child pairs living in the same geographic areas may share common characteristics concerning stunting and that some enumeration areas may have fewer observations raises concern of using BE-MGLMM. If this analysis is not used, we should expect underestimated standard errors, hence an increased likelihood of committing type one error [44]. Therefore, this study aimed to compare the SES indices in predicting stunting among under-five-year children in Tanzania accounting for correlation at regional and enumeration areas using the MLE-MGLMM and the BE-MGLMM.

## Methods

### Data source

The data used in this study were obtained from TDHS. The study used the 2004/5, 2010 and 2015/16 TDHS datasets being the recent surveys. The study used three surveys to determine the consistency of the constructed indices in predicting stunting. The DHS used a two-stage sampling technique to select households for study after stratifying the study areas into rural or urban in each region. The two-stage sampling started by selecting Primary Sampling Units (PSUs) from the most recent census enumeration areas (EAs). After selecting the PSUs from strata using the probability proportional to size, then a fixed number of households was selected from each PSU using equal probability systematic sampling. The detailed methodology for the DHS data collection and analysis was documented [49, 50].

The DHS collects information at the household level, on men and women of reproductive age who slept in the household a night before the survey. Of women aged 15–49 years, their children eligible for the study, their birth records and anthropometric measurements of both mothers and under-five children are taken. The unit of analysis for this study is a child–mother pair. The survey recruits women aged 15–49 years, and their respective under-five-year children are interviewed. The study merged the household member, household, individual and child recode datasets for each survey year using unique identifiers. The study inclusion criteria were children aged 0–59 months, while the exclusion criteria were children not living with their mothers, children with missing and out of range measurements of Height for Age Z-scores (HAZ) used for computing stunting status.

### Measurement of variables

The outcome of interest was stunting, classified as stunted if the child’s HAZ score was below–2.0 standard deviation (SD), otherwise not stunted [51]. The selection of independent variables was based on previous studies classified into three basic groups namely; child, maternal, and household covariates. Child covariates include sex, age (coded: 0–11 months, 12–33 and 34–49 months), size at birth (coded: small, medium and large), birth order [52], and self-reported diarrhoea status (coded as yes or no). A child was considered diarrheic if had loose stool more frequently than usual in two weeks Maternal characteristics included age [36], age at birth, breastfeeding duration, frequency of use of Antenatal Care (ANC) services, maternal BMI (coded: 0 “below 18.5” and 1 “at least 18.5” kg/m^2^) [52, 53], work status and age at first birth [52]. Household characteristics included the number of under-five children, household size, area of residence (rural or urban), and wealth index [54, 55]. The coding for each study variable is presented in appended Table 6.

**Table 1:** Participant’s background characteristics for selected socioeconomic variables

Variable	TDHS 2004/5 (*N* = 7492)		TDHS 2010 (*N* = 6806)		TDHS 2015/16 (*N* = 8929)	
	Unweighted	Weighted	Unweighted	Weighted	Unweighted	Weighted
	*n* (%)	%	*n* (%)	%	*n* (%)	%
Stunting						
No	4322 (57.7)	56.2	4109 (61.6)	59.7	5938 (67.5)	67
Yes	3169 (42.3)	43.8	2559 (38.4)	40.2	2852 (32.4)	33
Area of residence						
Urban	2401 (32)	40.5	1549 (23.2)	20.8	2007 (22.8)	26.1
Rural	5091 (68)	59.5	5119 (76.8)	79.2	6783 (77.2)	73.9
Wealth index						
Poorest	1217 (16.2)	15.7	1136 [17]	17.4	2032 [23.1]	24.5
Poorer	1037 (13.8)	9.7	1077 [16.1]	17.1	1830 [20.8]	21.7
Middle	1565 (20.9)	20.4	1424 [21.4]	20.9	1705 [19.4]	19.3
Richer	1542 (20.6)	21.4	1658 [24.9]	24.2	1813 [20.6]	18.5
Richest	2131 (28.4)	32.8	1373 [20.6]	20.4	1410 [16.1]	16
Mother’s education						
Uneducated	2088 (27.9)	26.4	1692 [25.4]	25.4	1909 [21.7]	21.3
Primary	4811 (64.2)	69.4	4213 [63.2]	68.4	5286 [60.1]	64.6
Secondary	593 (7.9)	4.2	763 [11.4]	6.2	1595 [18.2]	14.1
Mother’s work status						
Not working	1309 (17.5)	13.4	1118 [16.8]	13	1929 [21.9]	21.3
Currently working	6182 (82.5)	86.6	5545 [83.2]	87	6861 [78.1]	78.7
Mother’s BMI (kg/m^2^)						
Below 18.5	636 (8.5)	6.9	635 [9.6]	9.1	622 [7.1]	6.8
18.5–29.9	5642 (75.8)	78	4703 [70.7]	73.6	5895 [67.3]	68.5
25 +	1161 (15.6)	15.1	1309 [19.7]	17.3	2246 [25.6]	24.6
Household had < 5 years death						
No	2751 (36.7)	36.4	1775 [26.6]	27.2	1896 [21.6]	21.5
Yes	4741 (63.3)	65.6	4893 [73.4]	72.8	6894 [78.4]	78.5
Access to improved water						
No	5599 (74.8)	77.1	4904 [73.5]	80.3	6144 [69.9]	74.3
Yes	1887 (25.2)	22.9	1764 [26.5]	19.7	2646 [30.1]	25.7
Access to improved toilet						
No	7226 (96.5)	97.1	6231 [93.5]	94.6	7887 [89.7]	91.3
Yes	265 (3.54)	2.9	437 [6.5]	5.4	903 [10.3]	8.7
No. of < 5 years	2.03 ± 1.34		2.14 ± 1.25		2.12 ± 1.45	
HWI (overall)	− 6.97e-09 ± 2.04		− 1.05e-09 ± 2.12		− 5.59e-09 ± 1.95	
HWI (stunting)	0.02 ± 1.93		0.005 ± 1.51		0.003 ± 1.42	
WAMI	− 2.69e-09 ± 1.46		5.25e-09 ± 1.51		1.52e-09 ± 1.72	
WEO	− 5.75e-09 ± 1.94		− 2.13e-10 ± 1.85		− 1.87e-09 ± 2.11	
MPI	3.08e-09 ± 1.36		4.98e-09 ± 1.43		− 1.05e-08 ± 1.57	

No. of <5 years in a household and SES indices are reported using their mean ± standard deviation

The random variables included enumeration areas (EAs) and regions. The EAs are defined based on rural–urban areas perspective. In urban area, an EA may be a block or apartment building, while in rural areas it can either be a village or a group of villages. The DHS randomly selects EAs as adopted from the previous national census surveys. Based on regions, Tanzania had a total number of 26 regions by 2015. However, the composition of these regions in terms of geographical boundaries has been varying over time due to the changes in political regime and need for distribution of socioeconomic services. A detailed description on EAs and regions for each survey year are detailed [49, 50].

## Statistical analysis

### Descriptive analysis

The weighted and unweighted frequencies were presented in a frequency distribution table. The study used the Chi-square test of independence to determine the association between the selected socioeconomic categorical covariates and stunting status. The Pearson correlation coefficient was used to evaluate the relationship between constructed SES indices.

### Constructing SES indices

The household’s SES was constructed in four different indices namely the HWI, WAMI, WEO and MPI. These SES indices were constructed using the summated scores and principal component analysis (PCA) aided by the random forest (RF) technique for variable selection. The HWI was computed using PCA, while the WAMI, WEO and MPI were computed using both PCA and summated score. The RF was used to select the best eight wealth assets associated with stunting. Further description on computing SES indices using both summated score and PCA is presented.

#### Constructing SES indices using summated scores

The computation of WAMI, WEO and MPI includes independent variables like mother’s education, occupation, wealth assets and water and sanitation services. The mothers education was coded according to the UNESCO 2011 international standard classification of the level of education [56] while the occupation was classified according to the international standard classification of occupation [57] and upgraded relative to the importance of Tanzania. Occupation was grouped into four categories, namely; unskilled, semi-skilled, skilled and professional. The four categories were assigned weights ranging from 1 to 4, respectively [12]. Mother’s level of education was classified as uneducated, did not complete primary, completed primary, did not complete secondary, and completed secondary as well as completed higher education [12, 58]. These levels of education were assigned weights from 0 to 5, respectively [12]. Each wealth asset was coded as a binary variable, the RF was used to select the best 8 assets associated with stunting [13]. While other wealth assets were assigned a unit weight [59], car and land ownership were assigned weights 0.1 and 0.5, respectively [12]. Then, the summated score for the WEO index as adopted from [12, 59] was expressed as:

$$\mathrm{WEO}=\mathrm{wealth assets}+\mathrm{Education}+\mathrm{Occupation}$$

The second proxy indicator of SES was the WAM index, constructed using wealth assets, availability of improved water and toilets as well as maternal education. The availability of improved water and toilets formed the WASH index, which was then combined with maternal education and wealth assets. A household was termed having improved water and toilets according to WHO standards [60, 61]. The improved water and toilets were dummies thus each assigned a weight of four which then totaled 8 when forming the Water, Sanitation and Hygiene (WASH) score [13]. The maternal education was formed using years of education, the maximum being 16 years and then was divided by 2 to get the maximum score of 8. The best 8 assets associated with stunting were selected using the RF [13], then combined with WASH and maternal years of schooling to form the WAMI. Ever since DHS does not collect household income, this index did not consider income. Household income can be collected using actual income or proxy indicators like consumption and expenditure; however, its collection is difficult [59, 62] specifically in developing countries. Thus, the WAMI index adopted from [13] was expressed as: $\mathrm{WAMI}=\mathrm{WASH}+\mathrm{Assets}+\mathrm{Education}$.

Based on the MPI, each of the wealth assets ownership was coded as a dummy. A household was considered educated if either a mother is educated or a child attends school and was otherwise uneducated. Furthermore, whether a household had under-five death, as well as mother being undernourished were used. Child nutrition status being an indicator of MPI was not included because it was the dependent variable for this study. To reflect the poverty nature of MPI, the lack of ownership of a particular item was coded as 1 while owning a particular item was coded as 0. Therefore, the study expects the MPI to have opposite sign as compared to WAMI, WEO and HWI when predicting stunting.

#### Constructing SES indices using principal component analysis

The PCA is a variable reduction method used to identify correlated items, grouping them in similar constructs, thereby forming fewer dimensions or components [62]. Different studies used PCA in computing SES indices [62–64] for evaluating health problems. Normally, the first component has the largest eigenvalue representing the largest amount of variation in the original data [62]. Thus, the first component is a proxy of the particular index under consideration [62, 64]. For each SES index identified in the previous section, its corresponding items were expressed as dummy variables and then PCA was applied. Each SES index was modelled independently, and the first component was predicted and hence used for further analysis [62–64]. The obtained indices were used in their original forms, and no quintiles were developed.

### Statistical modelling

The statistical modelling of this study was centralized on the GLMMs. The GLMM typically use numerical optimization techniques to estimate model parameters and maximum likelihood estimation (MLE) is the most common method. Recently, the modelling of human health is featured in with the Bayesian estimation frameworks. This section provides a summarized explanation on the GLLMs and its corresponding estimation approaches, namely the MLE and Bayesian methods.

#### Generalized linear mixed models

The analysis of this study was centred on the generalized linear mixed models (GLMM) which extends the idea of generalized linear models (GLM). The GLM uses the linear predictor for the random and fixed effects under the assumption that the data are normal [65]. However, in some instances, the dependent variable is non-normal and the data have random effects, and then the GLMM are preferred [66]. Stunting status is a binary outcome following the Bernoulli distribution, and still child health characteristics are influenced the nested structure of EAs and regions hence requiring the use of binary logit mixed models which are under the umbrella of GLMM.

The study assumed that children born to the same mother, or children living with the same enumeration area (EA) or region may share common characteristics attributable to their stunting status. Thus, children may be nested within EA and EA may be nested within in regions. The nesting structure of children being nested to mothers formed level 1, the nesting structure of children being nested on EA formed level 2 while the nesting structure of regions formed level 3. A detailed expression on the named three models is presented under the specification of the mathematical models section.

#### Maximum likelihood approach

The MLE is one of the parameter estimation methods which utilizes the observed data to determine the parameter which maximizes the likelihood [67–69]. The parameters values are computed in a manner of being most likely to represent the observed data [67–69]. The MLE provides a simultaneous estimation approach of the fixed and random parameter [69]. However, the MLE works best in presence of larger sample sizes. In case of small sample sizes, the estimates are negatively biased [70] and sometimes the model may fail to converge.

In evaluating the parameter that maximizes the likelihood given the observed data, the MLE operates under two conditions; namely the first partial derivative known as the differential equation should be equal to zero and the second partial derivative should be less than zero. Mathematically, if we let $L\left[\theta /x\right]$ be the likelihood function,$\theta$; be the parameter value to be estimated from the observed data $x$, then the two conditions as adopted from [67] may be summarized as follows;

$$\frac{{\partial In L\left[ {\theta /x} \right]}}{{\partial y_{i} }} = 0\quad {1}^{{{\text{st}}}} {\text{condition}}$$

$$\frac{{\partial^{2} In L\left[ {\theta /x} \right]}}{{\partial y^{2}_{i} }} < 0\quad {2}^{{{\text{nd}}}} {\text{condition}}$$

The first condition aims at satisfying the existence of the parameter under MLE (${y}_{MLE}$ ${\uptheta }_{\mathrm{MLE}}$ ${\uptheta }_{\mathrm{MLE}}$) while the second condition aims at making sure that the ${y}_{MLE}$ ${\theta }_{\mathrm{MLE}}$ ${\theta }_{\mathrm{MLE}}$ is the maximum parameter value [67]. The MLE algorithm seeks to maximize the likelihood function, which measures how well the model explains the observed data.

#### Bayesian inference approach

The Bayesian model uses the prior distribution to update the posterior distribution when estimating the parameters. The prior information as obtained from the prior distribution represents the prior assumption about the parameter being estimated. The prior distribution influences the posterior distribution which represents the updated degree of uncertainty after analysing the particular data. If $X$ represents a random variable (stunting status) with the density function $f(x|\theta )$, the prior distribution with the density function $f(\theta )$; the density function $f\left(\theta |x\right)$ of the posterior distribution using Bayes theorem as adopted [71] may be computed.

$f\left(\theta |x\right)=\frac{f(x|\theta )f(\theta )}{\sum f\left(x|\theta \right)f\left(\theta \right)d\theta }$. Where $f(x|\theta )$ is defined as the likelihood function.

Normally, the posterior distribution is the product of the likelihood and the density of the prior distribution, expressed as $f\left(\theta |x\right)=L\left(\theta \right)f(\theta )$. Because stunting status follows the binomial distribution, the log link function was used when estimating the odds of stunting.

Based on the prior distribution, either the non-informative or the informative priors may be used [72, 73]. However, the informative priors may lead to biased estimates if not carefully chosen, or even obtained in sources where they are weakly defined [74] [75]. Additionally, compared to informative priors, non-informative priors provide a larger number of parameters to be estimated [76], and have minimum influence on the posterior distribution [71]. In such instances, the diffuse/non-informative prior are preferred [72]. The study used the flat diffuse normal priors with the distribution (0, 10,000) for fixed effect estimates and the inverse gamma priors for the higher hierarchical orders (hyperparameters) with the distribution (0.01, 0.01). In updating the posterior distribution, 10,000 simulations were conducted for each model at a burn-in of 2500.

#### The specification of mathematical models

The study estimated both the BE-MGLMM and MLE-MGLMM to assess the association between SES indices and stunting. The hierarchical nature of data was considered as children living in the same enumeration area or region may have the same characteristics towards acquiring stunting. Thus, children were nested in enumeration areas and enumeration areas were nested in regions. The standard logistic regression model without hierarchy, the two and three-level hierarchical model was constructed and compared. The hierarchy was based on the fact that enumeration areas were nested in regions. The three models namely the [1] standard logistic regression, [2] random effect model and [3] nested random effect model.

Model 1: $$\mathrm{log }\left({p}_{ij}/{1-p}_{ij}\right)={\beta }_{0}+ \beta X$$

Model 2: $$\mathrm{log }\left({p}_{ij}/{1-p}_{ij}\right)={\beta }_{0}+ \beta X+ {\mu }_{j}$$

Model 3: $\mathrm{log }\left({p}_{ijk}/{1-p}_{ij}k\right)={\beta }_{i}{X}_{k|j}+ {\beta }_{j}X+ {\mu }_{k|j}+ {\mu }_{j}$where $\mathrm{log }\left({p}_{ij}/{1-p}_{ij}\right)$ is the log odds of stunting for child $i$ living in region $j$. $\mathrm{log }\left({p}_{ijk}/{1-p}_{ijk}\right)$ is the log odds of stunting for child $i$ in region $j$ and enumeration area $k$. $X{\prime}s$ are the covariates. $\beta {\prime}s$ are the regression coefficient. ${\beta }_{j}X$ shows the extent to which region affects the slope of $X$. ${\beta }_{i}{X}_{k|j}$ shows the extent to which enumeration area affects the slope of $X$ in a given similar region. ${\mu }_{i}$ is the is a vector of variances for each random effect (regions) associated with stunting. ${\mu }_{k|j}$ is the random effect capturing showing the variation due to different enumeration area $k$ within a common region $j$. $X$ is the SES index under consideration.

The three models, namely level 1 (children nested in mother), level 2 (children nested in EAs), and level 3 (EAs nested in regions), were compared using Akaike information criterion (AIC) and the deviance information criterion (DIC) for MLE-MGLMM and BE-MGLMM, respectively. The model with three levels was revealed parsimonious due to lower AIC and DIC for MLE-MGLMM and BE-MGLMM, respectively. The Bayes prefix was used when conducting the B-MGLM. The obtained estimates for the B-MGLM were reported as Bayesian posterior odds ratio (BPOR) [77, 78] instead of the known posterior mean. The obtained results for the MLE-MGLMM were reported as odds ratio. Furthermore, the flexibility of Bayesian models to update the prior distribution ignores the need for sampling weights even for complex surveys [79, 80]. In this aspect, the study used unweighted samples. For the case of MLE-MGLMM, the Akaike information criterion (AIC) was used to decide on the best index predicting stunting. The rule of thumb is that the lower the AIC, the better the model. All analyses were conducted using STATA version 15.

## Results

### Participant’s demographic characteristics

The study revealed that out of 7492, 6806 and 8929 children, approximately 42.3%, 40.2% and 33% were stunted in 2004/5, 2010 and 2015/16, respectively. Of those who were stunted in 2010, 5219 (79.11%) lived in rural areas, 1699 (24.27%) lived in richer wealth quintile households, and 4290 (68.2%) were born to mothers with primary education. For the case of TDHS 2015/16, the study revealed that out of 2991 stunted children, 6911 (74.13%) were among those living in rural areas, 2069 (24.7) lived in the poorest wealth quintile households and 5368 (64.5) were among children born to mothers with primary education (Table 1).

Based on the distribution of stunting across the selected socioeconomic variables, the study revealed that the majority were 2129 [41.8], 1954 [38.2%] and 2363 [34.8] among children living in rural areas for survey years 2004/5, 2010 and 2015/16, respectively. The majority of stunted children were 956 [44.9%], 434 [40.3%] and 691 [37.8] among children living in the richest, poorer and poorer wealth quintile households for survey years 2004/5, 2010 and 2015/16, respectively. Furthermore, the study revealed that in all survey years, the majority of stunted children were 2667 [43.1%], 2212 [39.9%], and 2306 [33.6%] among children born to currently working mothers. A statistically significance difference between groups was observed for the mother’s education and the mother’s work status in all survey years. The rural–urban differences were statistically significant for survey years 2004/5 and 2015/16, while the wealth index was statistically significant for the 2015/16 survey (Table 2).

**Table 2:** Distribution of participant’s selected socioeconomic variables across stunting status

Variable	TDHS 2004/5			TDHS 2010		TDHS 2015/16	
	Stunted—yes		*p*-value	Stunted—yes	*p*-value	Stunted—yes	*p*-value
Area of residence							
Urban	1040 [43.3]			605 [39.1]		489 [24.4]	
Rural	2129 [41.8]		0.221	1954 [38.2]	0.53	2363 [34.8]	< 0.001
Wealth index							
Poorest	502 [41.2]			401 [35.3]		754 [37.1]	
Poorer	420 [40.5]			434 (40.3])		691 [37.8]	
Middle	650 [41.5]			549 [38.5]	0.108	634 [37.2]	< 0.001
Richer	641 [41.6]			657 [39.6]		491 [27.1]	
Richest	956 [44.9]		0.079	518 [37.7]		282 [20]	
Mother’s education							
Uneducated	867 [41.5]			707 [41.8]		709 [37.1]	
Primary	2111 [43.9]			1678 [39.8]		1786 [33.8]	
Secondary	191 [32.2]		< 0.001	174 [22.8]	< 0.001	357 [22.4]	< 0.001
Mother’s work status							
Not working	502 [38.3]			344 [30.8]		546 [28.3]	
Currently working	2667 [43.1]		< 0.001	2212 [39.9]	< 0.001	2306 [33.6]	< 0.001
Mother’s BMI (kg/m^2^)							
Below 18.5	257 [40.4]			286 [45]		233 [37.5]	
18.5–29.9	2426 [43]			1868 [39.7]		2036 [34.5]	< 0.001
25 +	463 [39.9]		0.089	398 [30.4]	< 0.001	577 [25.7]	
Household had < 5 years death							
No	1177 [42.8]			737 [41.5]		661 [34.9]	
Yes	1993 [42]		0.517	1822 [37.2]	0.001	2191 [8, 31]	0.011
Access to improved water							
No	2408 [43]			1985 [40.5]		2148 [34]	
Yes	759 [40.2]		0.035	574 [32.5]	< 0.001	704 [26.6]	< 0.001
Access to improved toilet							
No	3060 [42.3]			2467 [39.6]		2673 [33.9]	
Yes	108 [40.7]		0.606	92 [21]	< 0.001	179 [19.8]	< 0.001

### The association between SES indices and stunting

The association between SES indices and stunting was determined using the MLE-MGLM and B-MGLM as presented in Tables 3 and 4. Based on the AIC for selecting the best SES index in predicting stunting, the study revealed that MPI, HWI(overall) and MPI had lower AIC as compared to other indices for TDHS 2004/5, 2010 and 2015/16, respectively. For instance, the MPI had the lowest AIC of 11,567.5 as in 2004/5. In the 2010 survey, the HWI (overall) had a minimum AIC of 9757.9 in 2015/16, while the MPI had a minimum AIC of 11,919.1 as compared to other indices. The study observed a non-statistical significance association between SES indices and stunting hence the MPI having lower AIC was the best for the TDHS 2004/5. For the case of TDHS 2010, the lower AIC was for HWI (overall); however, the association was not statistically significant. For the case of 2015/16, all indices were statistically significant at *p* < 0.001, and the MPI having the lowest AIC was used (Table 4).

**Table 3:** The MLE-MGLM estimates of the relationship between SES indices and stunting

SES index	TDHS 2004/5			TDHS 2010		TDHS 2015/16	
	OR [95% CI]	AIC	OR [95% CI]		AIC	OR [95% CI]	AIC
HWI (overall)	0.99 [0.97, 1.01]	11,623.5	0.99 [0.97, 1]		9757.9	0.89 [0.87, 0.91]***	11,927.3
HWI (stunting)	0.99 [0.97, 1.01]	11,623.1	0.98 [0.95, 1]		9882.4	0.86 [0.83, 0.89]***	11,934.6
WAMI	1 [0.97, 1.02]	11,636	0.98 [0.95, 1.01]		9882.6	0.87 [0.84, 0.89]***	11,918.7
WEO	0.99 [0.97, 1.01	11,621.7	1 [0.98, 1.02]		9773.2	0.91 [0.88, 0.93]***	11,943.9
MPI	0.99 [0.96, 1.02]	11,567.5	1 [0.97, 1.04]		9780.7	1.13 [1.09, 1.16]***	11,919.1

***statistically significant at *p* < 0.001

**Table 4:** The BE-MGLMM estimates of the relationship between SES indices and stunting

SES index	TDHS 2004/5		TDHS 2010		TDHS 2015/16	
	BPOR [95% CI]	DIC	BPOR [95% CI]	DIC	BPOR [95% CI]	DIC
HWI (overall)	0.99 [0.97, 1.01]	11,587.6	0.99 [0.96, 1.01]	10,098.05	0.89 [0.87, 0.91]	12,310.66
HWI (stunting)	0.99 [0.97, 1.01]	11,589.5	0.98 [0.95, 1.01]	10,223.37	0.86 [0.83, 0.89]	12,311.39
WAMI	1 [0.97, 1.03]	11,601.6	0.98 [0.96, 1.01]	10,080.55	0.9 [0.87, 0.92]	12,309.1
WEO	0.99 [0.97, 1.01]	11,586.8	0.998 [0.97, 1.02]	10,113.2	0.91 [0.89, 0.93]	12,325.42
MPI	0.99 [0.96, 1.02]	11,534.7	1.04 [0.97, 1.11]	10,133.01	1.13 [1.1, 1.16]	12,290.38

*BPOR* Bayesian posterior odds ratio

Based on Table 3, the study revealed that each unit increase in MPI, the odds of stunting was 1% [OR = 0.99; 95% CI 0.96, 1.02] lower in 2004/5, however not statistically significant at even 5% level. For the case of 2010 TDHS, the study revealed that for each unit increase in HWI (overall), the odds of stunting declined by 1% [OR = 0.99; 95% CI 0.97, 1]; however, the association was not statistically significant even at a 5% level. In 2015/16, for each unit increase in MPI, the odds of stunting were statistically significantly increased by 1 [OR = 1.13; 95% CI 1.09, 1.16] at *p* < 0.001.

Based on the B-MGLM, we revealed that the MPI was the best predictor of stunting for the survey years 2004/5 and 2015/16, respectively. For the survey year 2010, the WAMI was the best index in predicting stunting due to its lower AIC as compared to other indices. As expected, the HWI, WAMI and WEO had a reducing effect on stunting for each unit score increase. Similarly, the MPI revealed an expected positive relationship with stunting, as each unit increase in MPI the odds of stunting increased (Table 4).

Compared to the MLE-MGLM, the B-MGLM had the capacity of producing variances for both the region and enumeration area random effects. The MLE-MGLM produced only variances for region-level random effects. However, results for variances pertaining to random effects were not reported in this paper. These estimates are available upon request.

From Tables 3 and 4, the study revealed that stunting was best predicted by the MPI for survey years 2004/5 and 2015/16, while the WAMI was the best for the TDHS 2010. Then, the multivariable analysis was conducted to oversee the behaviour of these indices adjusted for other covariates. Both the MLE-MGLMM and BE-MGLMM were used to model the association between the MPI and WAMI controlling for other variables. These variables included the child, maternal and household characteristics as described in Table 5.

**Table 5:** Multivariable analysis of the association between SES indices and CIAF

		Survey year	
Model/Factors	2004/5	2010	TDHS 2015/16
Model 1: MLE-MGLMM	AOR (95% CI)	AOR (95% CI)	AOR (95% CI)
A: Fixed factors			
MPI	0.99 [0.95, 1.03]		1.09 [1.04, 1.13]***
WAMI		0.37 [0.25, 0.54]***	
B: Random factors			
Variance (region)	0.064 [0.026, 0.155]	0.035 [0.014, 0.083]	0.0246 [0.0102, 0.0595]
Variance (enumeration area)			
Model 2: BE-MGLMM	BPOR (95% CCI)	BPOR (95% CCI)	BPOR (95% CCI)
A: Fixed factors			
MPI	0.99 [0.9, 1]		1.1 [1.1, 1.2]
WAMI		0.36 [0.3, 0.4]	
B: Random factors			
Variance (region)	0.07 [0.02, 0.14]	0.03 [0.01, 0.067]	0.03 [0.008, 0.075]
Variance (region > enumeration)	0.007 [0.003, 0.013]	0.007 [0.002, 0.013]	0.012 [0.003, 0.026]

Adjusted for

**Child characteristics:** age, sex, size at birth, diarrhoea status, birth type, birth orders, duration breastfed, type of delivery, fever, place of delivery. **Maternal characteristics:** age, age at first birth, marital status, BMI, media access, decision making, ANC attendant and ANC visits. **Household characteristics:** age of household head, sex of the household head, food security, total no. of under-fives, household size and residence area

*BPOR* means Bayesian posterior odds ratio, *CCI* means credible confidence intervals, *AOR* means adjusted odds ratio, *CI *means credible intervals

***represents statistical significance at < 1%

The study revealed that even after adjusting for other covariates, the effect of WAMI and MPI remained statistically significant for survey years 2010 and 2015/16, respectively. Still, stunting had a positive and negative association with the WAMI and the MPI, respectively. For instance, based on the results of the BE-MGLMM, the study revealed that for each unit increase in the WAMI, there was 64% [BPOR = 0.36; 95% CCI 0.3, 0.4] lower odds of a child to be stunted in 2010. Furthermore, for each unit increase in MPI, there was 1 [BPOR = 1.1; 95% CCI 1, 1.] times higher odds of a child to be stunted in 2015/16 (Table 5).

## Discussion

The study aimed to compare the SES indices in predicting stunting using Bayesian multilevel generalized mixed methods for TDHS 2004/5, 2010 and 2015/16. We formulated SES indices using PCA methods. These indices include the wealth assets index, the WAMI, WEO and MPI of which the latter is more complex as compared to others.

The study revealed that MPI had a better predictive capacity of stunting as compared to HWI, WEO and WAMI for the TDHS 2004/5 and 2015/16. For the case of TDHS 2010, the study revealed that WAMI had the best predictive capacity as compared to HWI, WEO and MPI. These findings that WAMI had the best predictive power are similar to a multicounty study conducted in Africa [13]. The fact that each score increases in WAMI results to lower odds of stunting is supported with ability of educated mothers to have better health-seeking behaviour, feeding practices and decision-making power [54, 81, 82]. The role of safe water and sanitation facilities in reducing contamination and eruption of diseases through repeated episodes of diarrhoea which further hinders child food intake and absorption [83] should be acknowledged. Furthermore, living in households with safe water and sanitation and educated mothers may imply higher purchasing power and hence access to nutritious foods which are the backbone for child growth.

It should be considered that WAMI and MPI are the same although works in a different direction and that MPI is more compact as it has additional variables like the mother’s nutrition status and having under-five death in a household. The findings that each score increase in MPI increases the odds of stunting are underlined with other studies which revealed that poor water and toilet facilities increase stunting [81, 84], poor maternal nutrition as measured by BMI [53, 85] and children born to uneducated mothers [30, 53, 81, 86–89] are prone to stunting. Furthermore, higher values of MPI imply the presence of poor water and toilet facilities which in turn are associated with communicable diseases like diarrhoea [83] as explained above.

Our findings are underlined by the fact that the observed statistical significance in variances of stunting might be attributed to the cultural aspect of educational achievement among mothers [13]. Working mothers were observed to have higher odds of stunting as compared to non-working mothers in previous studies [90] but contrary to [58, 91]. This might be attributed to the fact that the majority of working mothers do not spend much time with their children, as child rearing depends much on house girls and other relatives. It happens that a mother leaves the house at 05:00 AM and comes back home at 06:00 PM for those living in cities like Dar es Salaam and Mwanza. Unfortunately, house girls are normally young to the extent that they do not understand child feeding and caring practices. The assurance that a child is fed what the mother instructed remains doubtful. The observed differences in results compared to that of Ethiopia [91] might be due to cultural differences and food availability. For instance, compared to Tanzania, Ethiopia is largely a desert country with longer seasons of drought thus the majority of unemployed mothers are more likely to be undernourished together with their children due to lack of purchasing power and natural availability of food.

The departure of the constructed SES indices in predicting stunting underlies our hypothesis that these indices do not explain the same on health outcomes [13, 92, 93]. We have observed that the indicators forming a particular indices vary over time. The WEO has a combination of wealth assets, education and occupation. For instance, WAMI has a combination of wealth assets, water and sanitation services as well as maternal education while MPI is the more complex index. The complexity of MPI is defined by its nature of measuring the acute poverty [94].

This study did not bother about using maternal education as an indicator of SES alone as previous studies justified that multivariate indices are better than simple indices [12, 13, 15, 92]. The use of RF provided a more simplified index by selecting assets which are strongly associated with stunting was in agreement with other studies [13, 93, 95, 96]. The study by Krieger et al. [93] reported that rather than using education and occupation, income indicators were strongly associated with mortality, while the study by Psaki et al. [13] gave an assurance of using a multivariate WAMI index in predicting stunting after using RF methods for variable selection.

The study adopted the construction of the WAMI index from previous studies [13]. However, the index does not include household income as DHS does not collect this information. In case the income variable is not available, the use of maternal education, wealth assets as well as water and sanitation provided significant improvement in predicting stunting rather than using wealth assets or education independently [13].

Based on the methodological approach, the BE-MGLMM was revealed to be efficient when predicting the association between SES and stunting. This is due to the fact that compared to the MLE-MGLMM, the BE-MGLMM was capable of producing results for random effects. Furthermore, the compactness of 95% CCI was revealed to provide stronger estimates as compared to the MLE-MGLMM. These findings are underlined with the fact that the BE-MGLMM may provide unbiased estimates [48] evenly where the sample size is very small at hierarchical levels [46, 47].

### Conclusion

Stunting is still a major problem in Tanzania, varying significantly across regions and enumeration areas. The fact that individual child, maternal and household characteristics influencing child stunting are known still alarms us on the possibility of designing interventions which may reach each individual. This study revealed that contextual factors affects stunting among under-five-year children. The presence of contextual factors, attributable to social and cultural norms with respect to stunting, puts an emphasis on the need of designing well-targeted interventions being more specific to a particular region and enumeration areas as well. Compared to the WAMI index, which was the best predictor of stunting in the TDHS 2010, the MPI would be preferable when modelling stunting as it was the best predictor in 2004/5 and 2015/16. Initiatives should be embarked to make sure that safe water, sanitation facilities, maternal education and housing facilities are available to the socioeconomic disadvantaged population.

Furthermore, proper household wealth assets including dwellings should be of critical value to enhance a better living environment for under-fives. The pinpointed interventions need proper implementation which are bearable by the policy makers. Policies on budget re-allocation from the economic advantaged to the disadvantages population at lower levels like EAs are inevitable. The results of this study are in agreement with the parent–offspring conflict theory, the life history theory and the ecological model. This study emphasizes on other researchers to use these theories. Last but not least, the study emphasize the use of BE-MGLMM in modelling stunting controlling for contextual factors as compared to MLE-MGLMM. This is based on the fact that BE-MGLMM was able to produce random effects even at enumeration areas, believed to be unbiased estimates needed for programme and policy decisions.

The current study has several strengths, first having the capability of providing trustable estimates on the relationship between SES indices and stunting. This is because of having enough sample size and larger datasets obtained from TDHS which is generalizable to the whole country of Tanzania as compared to the results presented by Psaki et al. [13] which was the district level based study. Secondly, the study informs the reader that SES is truly a multidimensional aspect, and its effect on stunting varies over time. Instead of using HWI as the common measure of SES over time, corresponding SES index associated with the stunting should be established as SES indicators vary over time. However, based on these findings the MPI is recommended when assessing the determinants of stunting. Lastly, but not least, the study took into account the correlation between observations and quantified the presence of contextual factors associated with stunting through the use of both the MLE-MGLMM and BE-MGLMM. However, the BE-MGLMM was found more powerful to MLE-GLMM.

The findings of this study should be interpreted with caution as it is limited to some issues. Firstly, the findings should not be directly compared across surveys as some surveys did not have important variables needed for constructing the indices. For instance, the TDHS 2004/5 did not have information on land ownership, owning agricultural land and having a watch as well as a land telephone. In these aspects, the analysis was conducted on yearly bases. Secondly, these findings do not infer the causal relationship between SES indices and stunting. The DHS is normally cross-sectional in nature, thus we could not establish the causal relationship [97] between stunting and a set of independent variables under consideration. Lastly, but not least most of research questions like size of the child at birth, having fever and diarrhoea status are self-reported which increases the chance of obtaining biased estimates [98]. Furthermore, self-reported answers are prone to misclassification bias [99] however, have been trusted for different researches. Further studies should enlighten on three basic issues and these are (i) multilevel modelling of child wasting and underweight, as despite the observed lower prevalence at national levels, might be concealed within EAs and regional variability (ii) explore the spatial determinants of wasting and underweight, preferably using the Bayesian Spatial models which have been reported to identify the prevalent hotspots of a particular health outcome under consideration, and (iii) explore the spatial determinants of stunting and CIAF preferably using the Bayesian spatial modelling techniques, which are useful for identifying prevalent hotspots.

## Data Availability

The dataset is freely available from the DHS website at http://www.dhsprogram.com/data/dataset_admin/login_main.cfm.

## Appendix

See Table

**Table 6:** Description of study variables

Variable category	Variable description	Measurement level	Coding and labelling
*Dependent variable (s)*			
Stunting	A child was considered stunted if HAZ score was below -2SD, otherwise not	Dummy variables	0 “Not stunted” 1 “Stunted”
CIAF	A child was considered to have failure if he/she was suffering from any conventional indices of undernutrition, otherwise no failure	Dummy variables	0 “No CIAF” 1 “Had CIAF”
*Independent variable(s)*			
Child characteristics			
Sex	Whether a child is a male or female	Nominal	0 “Male”, 1 “Female”
Age	Age of a child in months	Categorical	0 “0–11”, 1 “12–23”, 2 “24–35”. 3 “36–47”, 4 “48–59”
Birth type	Whether birth was normal or caesarean	Nominal	0 “Normal” 1 “Caesarean”
Birth size	Whether size at birth was small, average or large	Ordinal	0 “Small” 1 “Average” 2 “Large”
Diarrhoea status	Whether a child had diarrhoea in three weeks or not	Dummy	0 “No” 1 “Yes”
Fever	Whether a child had fever or not	Dummy	0 “No” 1 “Yes”
Duration breastfed	Months a child was breastfed	Categorical	0 “ < 6” 1 “6–12” 2 “13–24” 3 “25 + ”
Twin	Whether a child was born as a twin or not	Dummy	0 “No” 1 “Yes”
Birth order	The order to which a child was born	Ordinal	0 “First–second”, 1 “third–fourth”, 2 “Fifth or more”
Mother’s characteristics			
Age	Age of the mother in years	Categorical	0 “15–24” 1 “25–34” 2 “35–49”
Marital status	Mother’s current marital status	Nominal	0 “ Single” 1 “Married” 2 “Separated/widowed”
Number of births in 3 years	Total number of births in 3 years	Ordinal	0 “None” 1 “Once” 2 “Twice or more”
BMI	Maternal BMI in kg/m^2^	Categorical	0 “ < 18.5” 1 “18.5 + ”
Decision making	Involved in making decisions on household purchases, visits and attending health services	Binary	0 “Not participate” if does not participate in any of household purchase, health care and household visits 1 Participates” if involved in any of the mentioned three items
Level of education	Mother’s level of education	Ordinal	0 “Not educated” 1 “Primary” 2 “Secondary” 3 “University”
Mother’s years of schooling	Number of years spent in school	Discrete	Any number from 0 to 16
Media access	Whether a mother reads magazines, listens to radio or watches television	Dummy	0 “No” if she does not read magazines, watch television or listen to radio; otherwise 1 “Yes”
Place of delivery	Whether a mother had a home or health facility delivery	Nominal	0 “Home” 1 “Health facility”
Skilled birth attendant	Whether a mother was attended by skilled personnel or not	Dummy	0 “No” 1 “Yes”
Prenatal service provider	Whether prenatal service was provided by skilled personnel or not	Dummy	0 “No” 1 “Yes”
Age at first birth	Mother’s age at first birth in years	Categorical	0 “ < 25”, 1 “25–30”, 2 “31 + ”
ANC visits	Number of ANC visits during pregnancy	Categorical	0 “ < 4 visits” 1 “At least 4 visits”
Household characteristics			
Household size	Number of people living in the household	Discrete	
Food insecurity	Problem in accessing food, not eating three meals a day, sleeping with hunger	Ordinal	0 “Never”, 1 “Sometimes/seldom”, 2 “Often/always”
Number of under-fives	Total number of under-five-year children in a household	Discrete	
Area of residence	Whether a participant lives in rural or urban area	Nominal	0 “Urban” 1 “Rural”
Household head’s sex	Whether a household is headed by a male or female	Nominal	0 “Male” 1 “Female”
Household head’s age	Age of household head in years	Categorical	0 “ < 20”, 1 “21–30”, 2 “31–40”, 3 “41–50″, 4 “”51 + ”
SES		Nominal	
Wealth assets	Household wealth assets ownership variable(s)	Binary	0 “Not own” 1 “Owns”
Mother’s education	Mother’s level of education	Ordinal	0 “Not educated” 1 “Primary” 2 “Secondary” 3 “University”
Mother’s work status	Whether a mother is currently working or not	Binary	0 “Not working” 1 “Currently working”
Child mortality	Household had < 5 years death	Binary	0 “No” 1 “Yes”
Water, sanitation and hygiene	Presence of improved water or toilets	Binary	0 “if water/toilets were not improved” 1 “if water/toilets were improved”

6.

## Abbreviations

AIC: Akaike information criterion
ANC: Antenatal care
BPOR: Bayesian posterior odds ratio
BE-MGLMM: Bayesian estimation multilevel generalized linear mixed models
DHS: Demographics and health surveys
DIC: Deviance information criterion
HAZ: Height for age Z-scores
HWI: Household wealth index
MPI: Multidimensional poverty index
PCA: Principal component analysis
MGLMM: Multilevel generalized linear mixed models
MLE: Maximum likelihood estimation
MLE-MGLMM: Maximum likelihood multilevel generalized linear mixed models
OR: Odds ratio
PSUs: Primary sampling units
RF: Random forest
SES: Socio-economic status
TDHS: Tanzania demographics and health surveys
WASH: Water, sanitation, and hygiene
WAMI: Water and sanitation, assets, maternal education and income
WEO: Wealth assets, education, and occupation
